# Impact of patient nationality on the severity of early side effects after radiotherapy

**DOI:** 10.1007/s00432-022-04505-0

**Published:** 2022-12-10

**Authors:** Mümtaz Köksal, Romy Streppel, Stefan Hauser, Alina Abramian, Christina Kaiser, Maria Gonzalez-Carmona, Georg Feldmann, Niklas Schäfer, Sebastian Koob, Mohammed Banat, Motaz Hamed, Frank A. Giordano, Leonard C. Schmeel

**Affiliations:** 1 Department of Radiation Oncology, University Medical Center Bonn (UKB), Bonn, Germany; 2 Department of Urology, University Medical Center Bonn (UKB), Bonn, Germany; 3 Department of Senology and Breast Center, University Medical Center Bonn (UKB), Bonn, Germany; 4 Department of Internal Medicine, University Medical Center Bonn (UKB), Bonn, Germany; 5 Department of Neuro-Oncology, University Medical Center Bonn (UKB), Bonn, Germany; 6 Department of Orthopedic Surgery, University Medical Center Bonn (UKB), Bonn, Germany; 7 Department of Neurosurgery, University Medical Center Bonn (UKB), Bonn, Germany; 8 Department of Radiation Oncology, University Medical Center Mannheim (UMM), Mannheim, Germany

**Keywords:** Immigration, Preventive medicine, Personalize therapy, Radiation therapy, Side effects

## Abstract

**Background:**

Major demographical changes in Germany commenced in the 1960s. Ongoing humanitarian crises in the Ukraine with subsequent immigration will have also long-range effects on national provision of cancer treatment. Ensuring the best possible outcomes for each cancer patient undergoing radiotherapy requires the prediction and prevention of unfavorable side effects. Given that recent research has primarily focused on clinical outcome indicators solely, less is known regarding sociodemographic predictors of therapeutic outcomes, such as patient nationality. Here, we investigated whether the severity of early side effects after radiotherapy are associated with patient nationality and other sociodemographic and clinical characteristics.

**Methods:**

Out of 9187 patients treated at a German university medical center between 2017 and 2021, 178 German and 178 non-German patients were selected for matched-pair analysis based on diagnostic and demographic criteria. For all 356 patients, data on side effects from follow-up care after radiotherapy were collected.

**Results:**

Non-German patients were more likely to have severe side effects than German patients. Side effect severity was also associated with tumor entity, concomitant therapy, body mass index, and age.

**Conclusion:**

Foreign cancer patients are at higher risk of experiencing severe side effects of radiotherapy, suggesting a need to develop and implement targeted preventive measures for these patients. Further research investigating factors predicting the occurrence of radiotherapy side effects, including other sociodemographic characteristics, is needed to better personalize therapy regimens for cancer.

**Supplementary Information:**

The online version contains supplementary material available at 10.1007/s00432-022-04505-0.

## Background

As the proportion of foreign medical patients in Germany increased from 2.7% in 2008 to 11.7% in 2020 (Destatis 2022), the incidence of oncological diseases has followed a similar trend. Considering the aging population, greater cancer risk with age, and improving cancer survival rate, the global incidence of cancer is expected to rise from ~ 9 million in 2017 to ~ 26 million by 2030 (Welzel and Tanner 2018; Eurostat 2019; Radkte 2022). Approximately 50% of cancer patients receive definitive or adjuvant radiotherapy (Verellen et al. 2007; Carlotto et al. 2013; Hayes et al. 2018; Delaney et al. 2005). Thus, given that foreign patients living in Germany are equally affected by this development, their use on radiotherapy as a component of their cancer treatment regimen is expected to increase in the future.

Curative radiotherapy aims to eliminate cancer stem cells while limiting damage to normal tissues (Barazzuol et al. 2020). Recent technological advances and clinical research have improved the ability of radiation oncologists to personalize radiotherapy parameters based on specific tumor and patient characteristics (Verellen et al. 2007; Baumann et al. 2016; Bernier et al. 2004). However, despite the use of optimized and state-of-the-art techniques, co-irradiation of peritumoral tissues is inevitable. Depending on the localization and dose of the ionizing radiation and affected organ sensitivity, a myriad of undesirable and extensive side effects may occur both early (≤ 3 months) and late (> 3 months) after treatment (Welzel and Tanner 2018; Lapierre et al. 2022; Ruysscher et al. 2019). Although not all patients are equally vulnerable to radiotherapy-related side effects, only a few avoid them (Sonis 2015). Hence, predicting which patients are most vulnerable to developing severe side effects of radiotherapy is important when personalizing treatment planning to prevent toxicity and improve the quality of life of cancer survivors (Lapierre et al. 2022).

The population of Germany is heterogeneous and dynamic, including an increase in the number of foreign citizens living in Germany over the past 10 years from 14.9 million to 22.3 million, representing ~ 27.3% of the total population (Destatis 2022). Apart from the continuous flow of immigrants into Germany since the 1960s, current events such as the humanitarian crisis in Ukraine also contribute to a changing population. Indeed, Eurostat forecasts a constant increase in net migration to Germany from 891,000 in 2015 to a peak of 1.37 million people by 2036 (Eurostat 2019).

Selecting the treatment regimen that is most beneficial to each patient requires consideration of the patient’s sociodemographic characteristics, clinical features, and genetic markers (Koenig et al. 2017; Sanzo et al. 2017; Conti et al. 2010; Tremblay and Hamet 2013). However, given the primary focus of recent research on predictive genetic markers (Ginsburg and Phillips 2018; McCarthy et al. 2013; Chin et al. 2011; Dzau et al. 2015; Golubnitschaja et al. 2016), limited information is available on the influence of sociodemographic characteristics on therapeutic outcomes. Concerning patient nationality, studies focusing on foreign cancer patients have evaluated disease incidence, treatment response, progression, and survival (Arnold et al. 2010; Budde 2020; Budde et al. 2019; Fischer et al. 2019; Hemminki et al. 2013; Mousavi et al. 2012; Rudiger et al. 2021; Spix et al. 2008; Thøgersen et al. 2018; Hjerkind et al. 2017) but have not sought to identify unique patient characteristics that predict beneficial or unwanted outcomes of therapy. Thus, a deeper understanding of the impact of sociodemographic characteristics on therapeutic outcomes, including unfavorable side effects, is needed to further personalize healthcare (England 2016).

The objective of this retrospective study was to investigate differences in the severity of early side effects of radiotherapy depending on patients’ nationality and other sociodemographic and clinical characteristics. Our findings show that foreign patients are at higher risk for severe side effects, suggesting the need to take patient nationality into account when planning treatment regimens for cancer patients to minimize unwanted side effects and improve their quality of life.

## Materials and methods

A total of 9187 patients were documented in our university medical center records between January 2017 and December 2021 as having received radiotherapy. Based on information stored on patients’ eHealth Cards, 8651 patients had German nationality and 536 patients had non-German (i.e., foreign) nationality. Two patients were incorrectly identified as German on their eHealth Cards as evidenced by having addresses at the embassy of the United Arab Emirates. After reclassifying these patients, 8649 and 538 patients had German and foreign nationality, respectively. A total of 289 foreign patients were excluded due to having no diagnosis recorded in our medical records, 43 foreign patients were excluded for receiving radiotherapy at a different institution, and 7 foreign patients were excluded because their nationality was unclearly filed.

Matched-pair analysis was conducted to match each included foreign patient with a German patient at a 1:1 ratio based on age (± 15 years), sex, and ICD-10 diagnosis code. If all matching criteria besides ICD-10 code corresponded between patients, ICD-O-3 classification was used for matching (WHO 2019). This ICD-O-3 matching approach was used for 10 matched pairs (i.e., 20 patients). Eight matched pairs shared the same ICD-O-3 code of M8070/3 [squamous cell carcinoma, not otherwise specified (NOS)]. Of the 16 patients included in these eight matched pairs, 14 were assigned an ICD-10 subchapter code of C00-C14 (malignant neoplasms of lip, oral cavity, and pharynx), 1 was assigned a subchapter code of C76 (malignant neoplasm of other and ill-defined sites; localized cervically), and 1 was assigned a chapter code of C80 (malignant neoplasm without specification of site; localized cervically). Regarding the remaining two ICD-O-3-matched pairs, one pair had divergent ICD-10 codes of C18 and C21 (malignant neoplasm of colon/anus and anal canal) but the same subchapter code (malignant neoplasm of digestive organs) and radiation site (rectum and pelvic lymphatic drainage area) as well as corresponding ICD-O-3 code M8140/3 (adenocarcinoma, NOS). In the other matched pair, both patients presented with leukemia [lymphoid (C91) and myeloid (C92) leukemia, respectively], corresponding to the ICD-O-3 code M9801/3 (acute leukemia, NOS). Given the insignificance of these discrepancies, we proceeded with analysis.

**Fig. 1:**
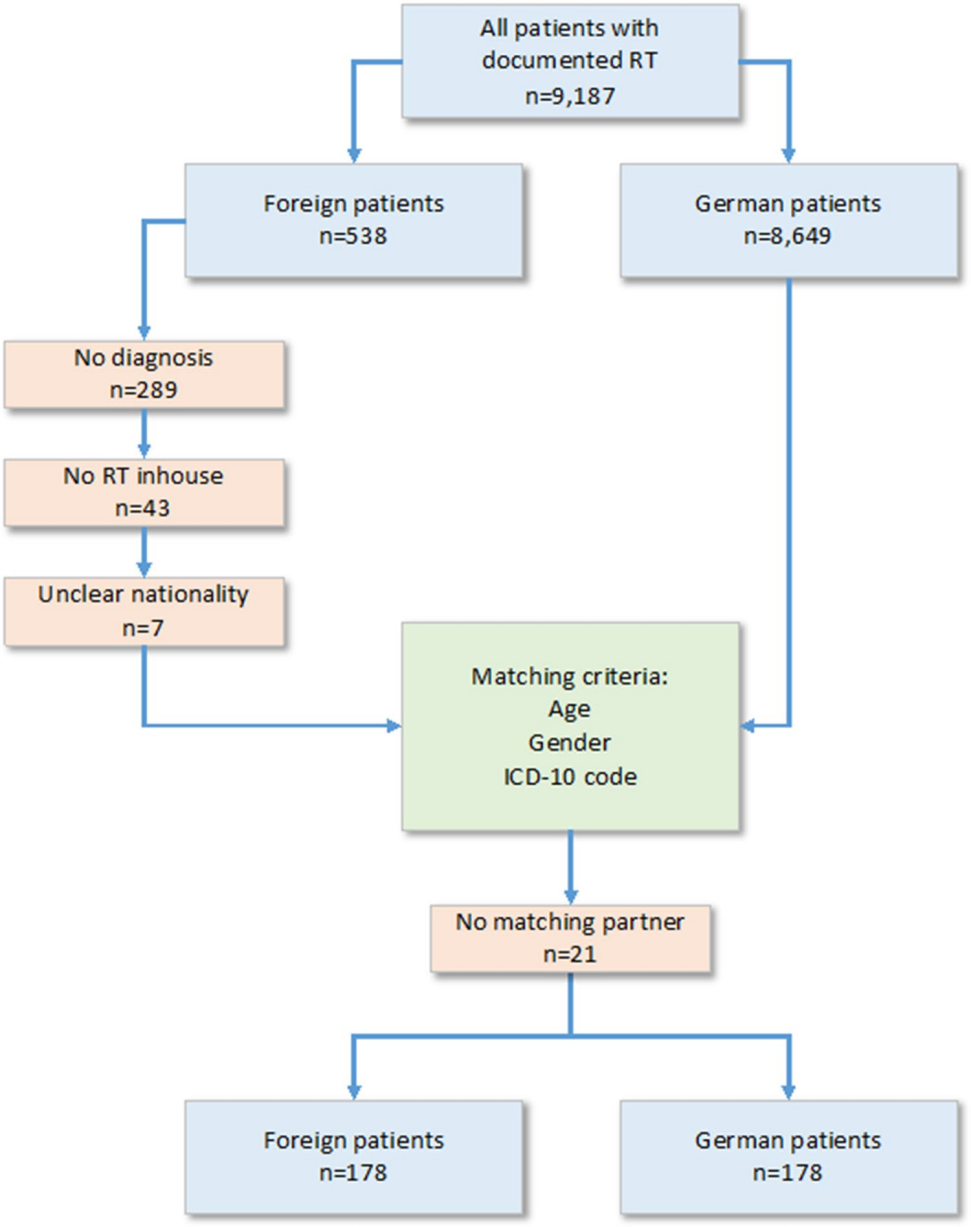
Data collection and patient matching process. RT, radiotherapy

After excluding 21 foreign patients for whom no suitable matched German partner was found, a total of 178 matched pairs were included in the analysis (Fig. 1). Thus, the total cohort consisted of 356 patients. An overview of all collected parameters is shown in Table 1 (Supplementary Materials).

Side effects were defined as “any unfavorable and unintended sign, symptom, or disease temporally associated with the use of a medical treatment or procedure” according to the Common Criteria for Adverse Events (CTCAE, version 5.0) of the National Cancer Institute (US Department of Health and Human Services 2017). Early side effects (≤ 3 months after radiotherapy) experienced by patients were identified by CTCAE terms grouped into System Organ Classes according to Medical Dictionary for Regulatory Activities hierarchy (ICH 2022). Example classes included cardiac disorders, general disorders and administration site conditions, immune system disorders, and nervous system disorders. The grading scale for side effect severity ranged from 1 for asymptomatic or mild symptoms to 5 for death related to side effects. Data on side effects was obtained via systematic search of patients’ medical records and collection of clinically relevant entries in the ARIA^®^ system (i.e., documentation of patient rounds, correspondence by email or telephone, physician letters, final and follow-up reports). Side effects were classified into CTCAE categories by assistant or specialist physicians or by the researchers based on medical record documentation. Comorbidities were assessed using the Charlson Comorbidity Index (CCI). Scores were based on the number of comorbidities, each given a weighted integer from 1 to 6 depending on its severity (Austin et al. 2015).

IBM SPSS^®^ (Chicago, IL) for Mac (version 28.0.1.1) was used for statistical analyses. Pearson’s Chi-square tests were used to evaluate associations among nationality, other patient characteristics, and side effect severity. Following a significant Kolmogorov–Smirnov test (*p* < 0.01), a Mann–Whitney *U* test was used to determine whether the number of side effects differed between German and foreign patients. All tests were two sided, with *p* < 0.05 indicating statistical significance.

## Results

### Patient characteristics

Both the German and foreign patient groups included 87 males and 91 females (Table 2, Supplemental Materials). Mean age was 56.1 years (range 2–85 years) among German patients and 55.9 years (range 3–89 years) among foreign patients. A total of 138 patients (38.8%) had a primary tumor, 174 (48.9%) had secondary manifestations (i.e., metastases), and 44 (12.4%) had disease relapse. Most patients received radiotherapy alone (*n* = 216, 60.7%) or combined chemoradiotherapy (*n* = 112, 31.5%). Foreign patients were significantly more likely than German patients to need an interpreter to provide informed consent.

**Fig. 2:**
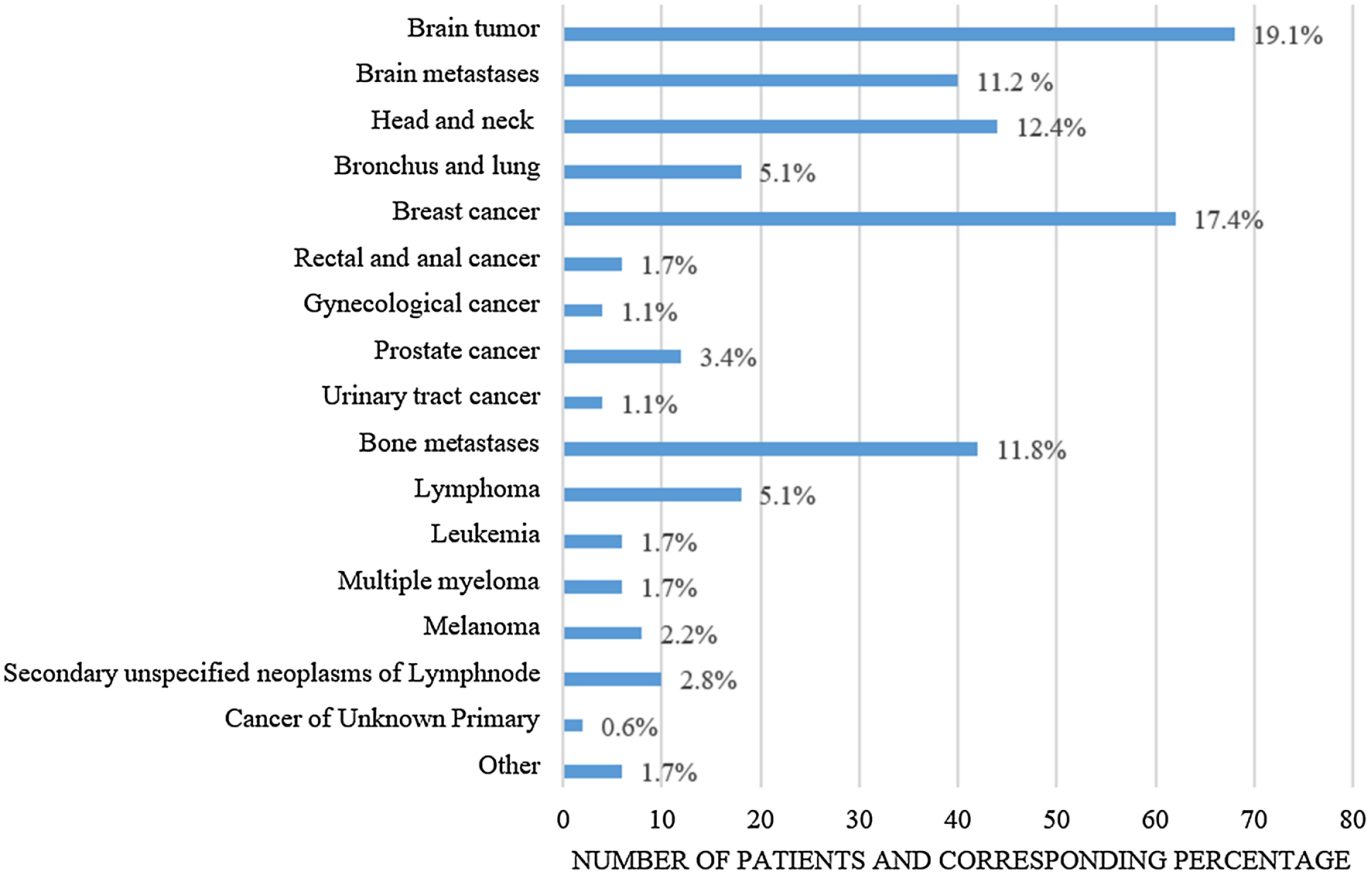
Frequencies of tumor entities categorized into 17 superordinate groups

Across both patient groups, significantly more patients had solid tumors (*n* = 326, 91.5%) than malignant hematologic diseases (*n* = 30, 8.5%) (*p* < 0.001). Brain tumors were the most common tumor entity (*n* = 68, 19.1%), followed by breast carcinomas (*n* = 62, 17.4%) and head and neck tumors (*n* = 44, 12.4%) (Fig. 2 and Table 3, Supplemental Materials).

**Fig. 3:**
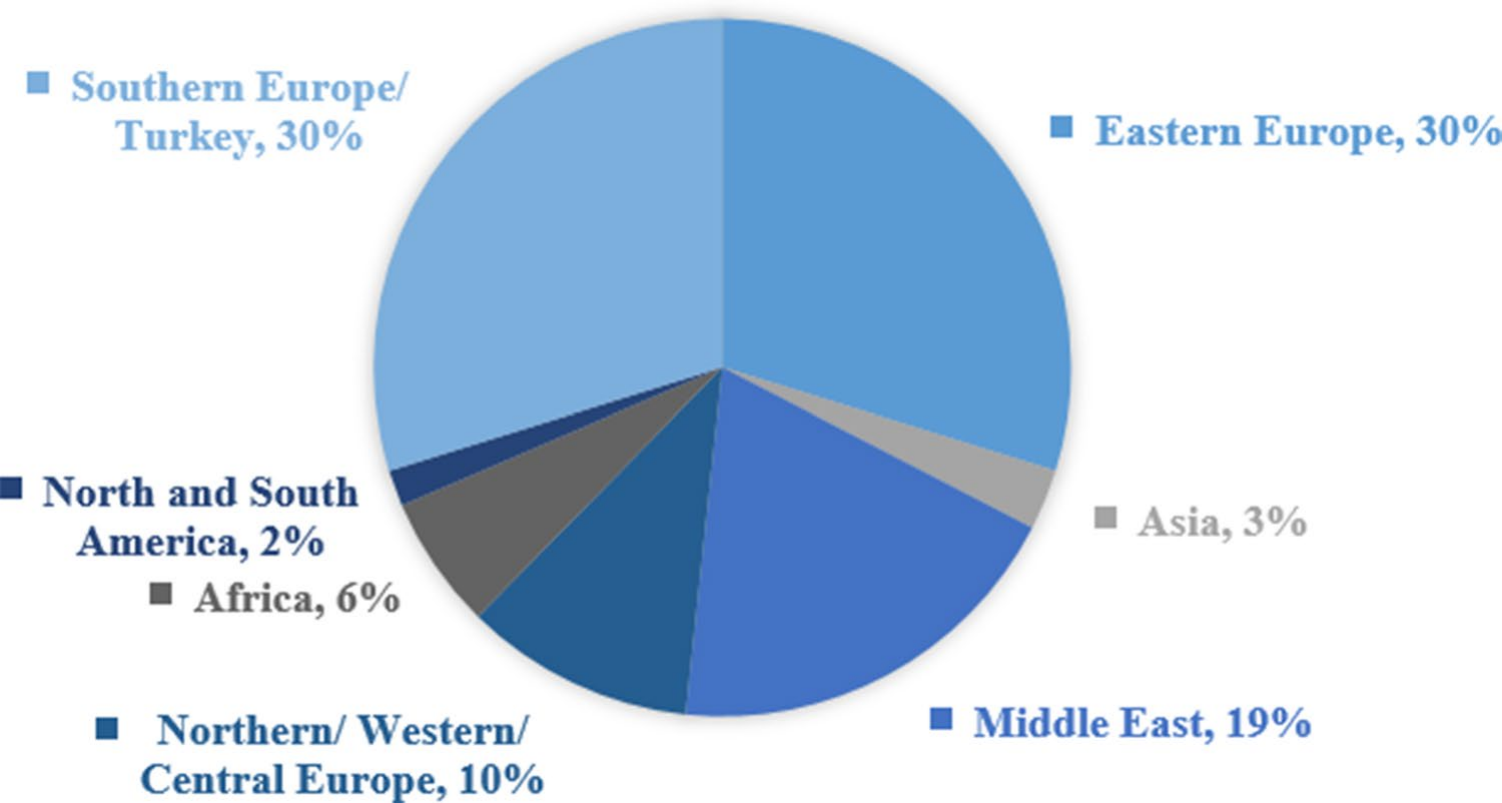
Origin of foreign patients categorized by geographic region

Foreign patients had a total of 53 different nationalities, which were grouped into seven regions (Fig. 3). Most patients were from Eastern European (*n* = 53), Southern European/Turkish (*n* = 53), or Middle Eastern (*n* = 34). Regarding specific countries of origin, foreign patients were most frequently from Turkey (*n* = 28; 15.7%), Russia (*n* = 13; 7.3%), Italy (*n* = 12; 6.7%), or Saudi Arabia (*n* = 10; 2.8%) (Table 4, Supplemental Material).

### Frequency and severity of side effects

German patients experienced a total of 776 side effects, whereas foreign patients experienced a total of 865 side effects (Table 1). Patients were categorized into mild-to-moderate or severe side effect groups based on the maximum severity of all side effects experienced. The mild-to-moderate group had a maximum severity of 0 or 1 for at least one side effect, whereas the severe group had a maximum severity of 2 or 3 for at least one side effect. Less than half of German patients (*n* = 75, 41.2%) and more than half of foreign patients (*n* = 98, 55.1%) were categorized into the severe side effect group. After calculating weighted score sums (i.e., mean severity across all indicated side effects), German and foreign patients had mean severity scores of 1.18 and 1.25, respectively.

**Table 1:** Frequency of side effects among German and foreign patients

Severity	German patients (*n* = 178)	Foreign patients (*n* = 178)
1	650	683
2	111	150
3	15	32
Total	776	865

Eleven German patients and six foreign patients did not report any side effects

**Fig. 4:**
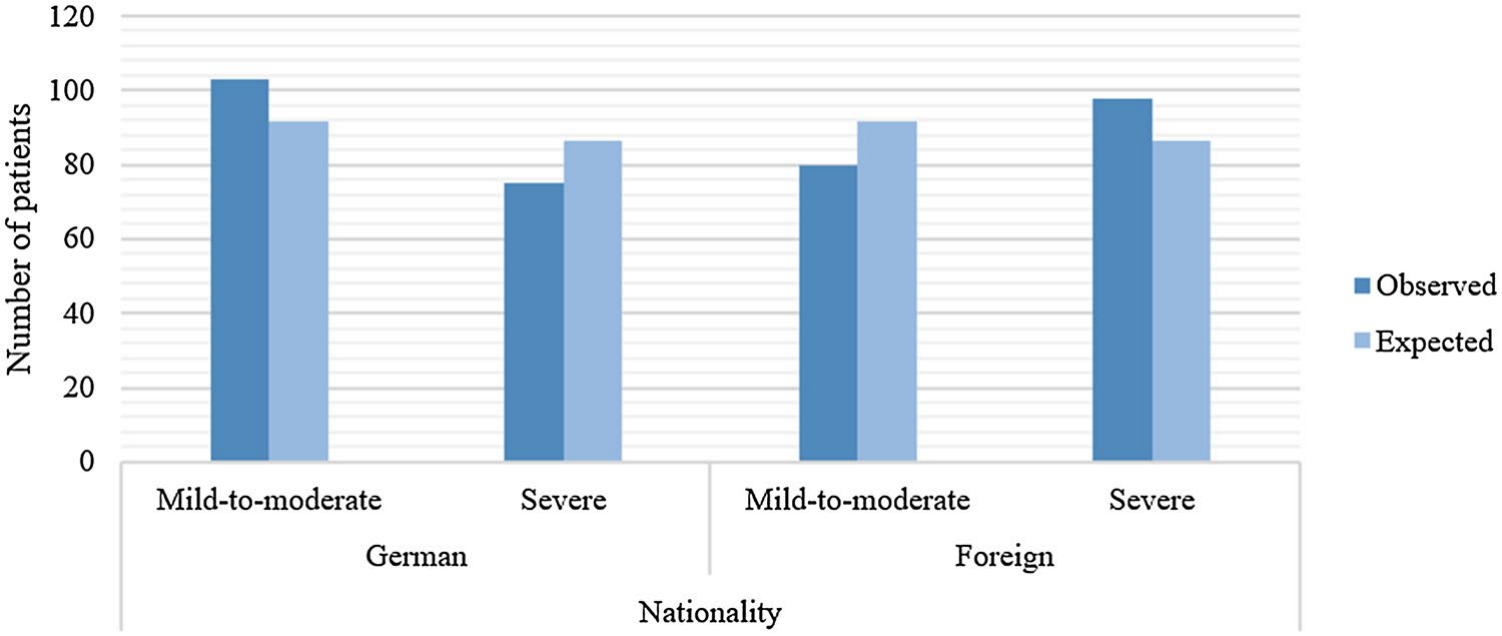
Expected and observed numbers of German and foreign patients who experienced mild-to-moderate and severe side effects

Side effect severity was significantly associated with patient nationality [*χ*^2^(1) = 5.949; *p* < 0.05; *φ* = 0.129] (Fig. 4). More foreign patients experienced severe side effects than expected (98 vs. 86.5), whereas more German patients experienced mild-to-moderate side effects than expected (103 vs. 91.5). However, there was no significant difference in the number of side effects experienced by German and foreign patients (*U* = 14,609.50; *Z* = − 1.277; *p* = 0.202).

Side effect severity was also significantly associated with tumor entity [*χ*^2^(16) = 66.964; *p* < 0.01; *φ* = 0.434] and concomitant therapy [*χ*^2^(3) = 15.469; *p* < 0.01; *φ* = 0.208]. Regarding tumor entity, more patients with head and neck tumors experienced severe side effects than expected (42 vs. 21.4), whereas more patients with bone metastases experienced mild-to-moderate side effects than expected (27 vs. 21.6). Regarding concomitant therapy, more patients receiving combined chemoradiotherapy experienced severe side effects than expected (70 vs. 54.4). However, in the tumor entity and concomitant therapy analyses, 17 and 2 cells had a frequency of < 5, respectively, which could potentially cause erroneous results, although results of post hoc Fisher’s exact tests and the Monte Carlo method verified these results (both tests, *p* < 0.001). There were no associations between side effect severity and CCI, smoking, or alcohol consumption.

Among foreign patients, side effect severity was significantly associated with body mass index (BMI) [*χ*^2^(3) = 7.917; *p* < 0. 05; *φ* = 0.211]. More patients with a BMI category of 3 or 4 experienced severe side effects than expected (BMI 3 = 37 vs. 29.7, BMI 4 = 15 vs. 13.8), whereas more patients with a BMI category of 1 or 2 experienced mild-to-moderate side effects than expected (BMI 1 = 9 vs. 6.3; BMI 2 = 44 vs. 38.2). Finally, among Turkish patients, side effect severity was significantly associated with age (< 60 vs. ≥ 60 years) [*χ*^2^(1) = 7.337; *p* < 0.01; *φ* = 0.512]. Similar associations were not observed within other regional or country patient subgroups.

## Discussion

We investigated whether German and foreign cancer patients differed in the quantity and severity of early side effects experienced following radiotherapy. Although we found no difference in the number of side effects, we found that foreign patients were more likely to experience severe side effects than German patients. Side effect severity was also associated with tumor entity and concomitant therapy, with patients who had head and neck tumors or received combined chemoradiotherapy being more likely to experience severe side effects. Among foreign patients, a higher BMI was associated with a greater risk of severe side effects. Furthermore, among Turkish patients, older patients (≥ 60 years) were more likely to experience severe side effects than younger patients (< 60 years).

Previous studies suggest that multiple factors are associated with poorer therapeutic outcomes among foreign patients than among national patients. For instance, foreign people struggle with structural access barriers to health services (e.g., fees, waiting times, travel distances). These barriers could be partially addressed by providing newcomers with easy-to-understand information on the availability and accessibility of healthcare services available in Germany or developing healthcare apps that promote adherence to therapy and allow the identification of new or worsening side effects (Klein and Knesebeck 2018; Starker et al. 2021; Academy of Medical Sciences 2007). Language barriers also remain a problem that undermines the accessibility and quality of healthcare services provided to foreign patients and are associated with limited access to health information and patient education, thereby exaggerating social inequities in knowledge regarding health issues (Rechel et al. 2013; Mosdøl et al. 2018). Encouraging patient involvement in treatment choices, which enhances compliance, requires providing patients with useful information about therapy options and their risks of side effects (Sonis 2015). In our study, all foreign patients received a German version of a patient education brochure titled “Thieme Compliance for Radiotherapy”; however, only 43 of 178 foreign patients (24.2%) had a personal interpreter present during discussions with a healthcare provider about treatment plans. To address this problem, patient education should be provided in the patient’s native language or should make use of pictures and diagrams to facilitate understanding (Rechel et al. 2013). Also, trained interpreters provided by the healthcare institution should be used to ensure adequate communication and clarification of questions and uncertainties (Thornton et al. 2009).

In line with the previous findings that foreign patients have inherently poorer health behaviors and inaccurate perceptions about the impact of health behaviors (Liu et al. 2019), we found an association between BMI and side effect severity among foreign patients. This result suggests that targeted outreach to foreign patients could be used to provide education on healthy nutrition and the benefits of moderate exercise, which may be particularly effective when performed by “ethnic health educators” in patients’ native languages (Fernandes and Pereira-Miguel 2009). Foreign patients also show less willingness to participate in screening and prevention programs and poorer compliance with follow-up examinations (Klein and Knesebeck 2018). Consistently, we also found that foreign patients had a higher rate of treatment termination and lower rate of attendance at follow-up appointments compared with German patients, although these differences were not statistically significant (Table 2, Supplemental Material). To improve patients’ participation in their own healthcare, active, patient-centered follow-up programs should be established to improve therapeutic outcomes and allow better evaluation of the effectiveness and safety of radiotherapy at the population level (Ruysscher et al. 2019; Andreyev 2007).

Acting upon these findings is important not only for improving clinical care and patient outcomes but also for the field of health economics, which plays an increasing role in healthcare decision-making (Sonis 2015). The economic burden of acute toxicities associated with cancer treatment has long been recognized (Carlotto et al. 2013), with mounting direct costs of managing side effects such as medication, hospitalization, and use of physiotherapists or psycho-oncologists. Furthermore, as side effects carry the risk of eventually becoming chronic, thereby prolonging rehabilitation and incapacity for work (Diz Dios and Diniz Freitas 2020; Schmielau et al. 2017), indirect costs such as loss of opportunity, work time, and productivity and increased need for caregiver support add to the already heavy economic burden on society. Therefore, preventing side effects of radiotherapy is economically advantageous to treating side effects after they occur (Carlotto et al. 2013). By identifying patients in which severe side effects are most likely to occur, unwanted outcomes of cancer therapy can be prevented, leading to better symptom control and quality of life (Ruysscher et al. 2019).

In addition to patient nationality, we also found an association between tumor entity and side effect severity, such that patients with head and neck cancer were more likely to experience severe side effects. These patients are especially prone to developing side effects given that they are at high risk for malnutrition due to their cancer site, disease process, and treatment. Detrimental lifestyle factors associated with the development of head and neck cancers, such as alcohol misuse, also increase patients’ risk for severe side effects (Talwar et al. 2016). Therefore, the prevention and treatment of side effects in patients with head and neck cancers remains a challenge (Nigro et al. 2017). However, as our subgroup analysis included only 44 patients, further evaluation of the risk of early side effects among head and neck cancer patients requires studies with larger sample sizes and more detailed analyses of different types of side effects and potentially predictive characteristics.

Consistent with previous studies, we found that patients receiving combined chemoradiotherapy had a higher risk of developing severe side effects than patients receiving neoadjuvant chemotherapy. Two reasons for these findings must be considered: (1) concurrent chemotherapy increases tissue sensitivity to radiation damage, and (2) neoadjuvant chemotherapy, which decreases the size of the primary tumor, results in a lower total radiation dose to nearby organs (Yao et al. 2017). Thus, when personalizing treatment planning for each cancer patient, a balance between treatment efficacy and potential for side effects must be achieved so that patients receive the greatest possible benefit from therapy with the least potential for side effects.

Among patients from Turkey, a generation effect was found such that older patients (≥ 60 years), many of whom may have been first-generation foreigners who migrated to Germany in the 1960s, were more likely to experience severe side effects than younger patients (< 60 years). Consistent with our findings, previous studies report differences in treatment outcomes and side effects within migrant populations depending on whether they are first-generation migrants who immigrated to the host country themselves or second-generation migrants who were born in the host country (Starker et al. 2021). However, other studies found no effect of migrant status on health (Wengler 2011). These discrepant findings may be due to the existence of confounding factors, such as socioeconomic status and education, language skills, social status, lifestyle, work status, and participation in society, which greatly impact overall health and therapeutic outcomes, including side effects (Weber and Hörmann 2011). Importantly, our finding that foreign patients were more likely to experience severe side effects than German patients was no longer observed when restricting the analysis to patients < 60 years of age [*χ*^2^(1) = 2.687; *p* = 0.101; *φ* = 0.119]. One reason for this finding could be that first-generation immigrants were less informed about the German healthcare system and less fluent in the German language compared with second-generation immigrants, thus leading to less active participation in the healthcare system (Glaesmer et al. 2011). This finding underlines the need to focus on preventing side effects among first-generation immigrants receiving radiotherapy.

Our work contributes to efforts to prevent disease and negative health outcomes among patients who are at higher risk due to personal characteristics, which has generated global policy interest (Dzau et al. 2015). In contrast to other studies on this topic, the present study obtained and analyzed a large number of potentially confounding variables, such as comorbidity, alcohol use, smoking, and BMI. Despite these strengths, however, our study also has several limitations. Due to the retrospective nature of our study, causal relationships cannot be determined. The method of data collection was prone to transcription errors. Also, our analysis included 17 groups of patients with different tumor entities, with some groups being quite small, leading to heterogeneity in the data and limiting statistical power. As such, studies with larger sample sizes are warranted.

A further limitation of our study is how patient nationality was determined. We categorized patients based on information stored on their eHealth Cards and grouped all patients with foreign passports into a single category. Due to the retrospective nature of the study, we did not have information about whether patients were economic migrants, students, return migrants, refugees, retirement migrants, or medical tourists, the latter of which show large variations in tacit knowledge about medical treatment and healthcare systems (Ormond 2016). We also could not determine whether migrants were born and raised in Germany, as second- and third-generation foreigners often do not have German citizenship owing to various reasons (Fick 2016). In addition, we did not assess foreign patients’ language skills, length of stay in Germany, or legal status, which are associated with use of preventative care and healthcare services as well as overall health (Starker et al. 2021; Dias et al. 2008; Acevedo-Garcia et al. 2010; Lebrun 2012). Therefore, more detailed assessment of patients’ migration background would enable future research to consider the extensive diversity in socioeconomic, political, and legal statuses of people pursuing and receiving medical treatment abroad (Starker et al. 2021; Ormond 2014).

Finally, information on side effects was collected in a subjective manner by clinicians or generated by the researchers based on information contained in medical center records. This approach is prone to error and can underestimate the incidence and severity of symptomatic side effects, thus reducing sensitivity and specificity (Fromme et al. 2004). Therefore, employing a patient-centered survey of side effects, such as the Patient-Reported Outcomes Version of the CTCAE, may provide a more accurate picture of patients’ experiences of side effects as well as subjective burden and health-related quality of life (Fromme et al. 2004; Greimel et al. 2011) and thereby enable better prediction of unfavorable clinical events (Basch et al. 2009).

## Conclusions

Acute side effects occur after nearly all types of non-surgical cancer interventions are dose-limiting, reduce patients’ quality of life, and contribute to the economic burden of disease and healthcare costs. Seeking to improve the prediction of acute side effects, we identified foreign patients as being more vulnerable to severe side effects of radiotherapy than German patients. This finding suggests that systematic, targeted preventive and supportive measures should be incentivized for foreign patients to enhance their therapeutic outcomes and improve their quality of life. These measures must not necessarily be innovative considering that the simple adaption and improvement of existing clinical processes and treatment approaches can have great value. In addition, existing healthcare information and patient education programs should be tailored for foreign patients considering differences in their needs and preferences.

## Supplementary Information

Below is the link to the electronic supplementary material.

Supplementary file1 (PDF 241 KB)

## Data Availability

The data that support the findings of this study are available from the corresponding author upon reasonable request.
