# Supplementary material for: Impact of patient nationality on the severity of early side effects after radiotherapy
Source: J Cancer Res Clin Oncol. 2022 Dec 10;149(9):5573–82. doi: 10.1007/s00432-022-04505-0 (PMC10356627; doi:10.1007/s00432-022-04505-0)
Supplement: Supplementary file 1 — Supplementary file1 (PDF 241 KB) [file 432_2022_4505_MOESM1_ESM.pdf]

Supplemental Materials

Table 1 Overview of all collected variables

Demographic variables	Name, patient ID
Patient characteristics	Body mass index, smoking, alcohol, supportive therapy, medication, Charlson Comorbidity Index, Karnofsky Index
Therapy	Duration of radiotherapy (RT), concomitant therapy [RT alone, RT with chemotherapy (CTx), RT with immune/hormone therapy (ImTx/HTx)], pre/post therapy administered [RT, CTx, ImTx, HTx, surgery, stem cell transplantation (STx), radionucleid therapy], therapy goal (definitive, adjuvant, palliative, pre-STx), dose (Gy), irradiation site, break RT, termination RT
Diagnosis	Date of first diagnosis, tumor entity, tumor status (primary, secondary, relapse), and tumor grading
Others	Informed consent (alone/with relatives/with interpreter), language of informed consent, treatment setting (outpatient/inpatient), follow-up received, number of patient rounds
Side effects	Number, and severity of side effects (CTCAE v5.0)

Table 2 Patient characteristics

**All patients (n = 356)**	**Foreign (n = 178)**		**German (n = 178)**				
	**n**	**%**	**n**	**%**	**Chi-square**	**Mann-Whitney U**	**p-value**
Age (years)							
<60	93	52.2	98	55.1			
≥60	85	47.8	80	44.9	0.595		0.282
Mean	56.1		55.9			15686.5	0.873
Median	58.5		58.0				
Range	Min. = 3, Max. = 89		Min. = 2, Max. = 85				
Sex							
Male	87	48.9	87	48.9			
Female	91	51.1	91	51.1			
Tumor status					0.801		0.444
Primary	66	37.1	72	40.4			
Secondary	89	50.0	85	47.8			
Relapse	23	12.9	21	11.8			
Concomitant therapy					0.856		0.774
Radiotherapy alone	111	62.5	105	59.0			
Chemoradiotherapy	55	30.9	57	32.0			
Immunotherapy	9	5.1	12	6.7			
Hormone therapy	3	1.7	4	2.2			
Radiotherapy goal					0.834		0.866
Definitive	32	18.0	38	21.3			
Adjuvant	85	47.8	80	44.9			
Palliative	58	32.6	58	32.6			
Pre-stem cell transplantation	3	1.7	2	1.1			
Treatment setting					1.071		0.585
Outpatient	112	62.9	114	64.0			
Inpatient	66	37.1	64	36.0			
Treatment termination					1.544		0.214
No	167	93.8	172	96.6			
Yes	11	6.2	6	3.4			
Informed consent					55.348		<0.001
No information	34	19.1	66	37.1			
With relatives	100	56.2	108	60.7			
With relatives and interpreter	23	12.9	0	0			
Without relatives	1	0.6	4	2.2			
With interpreter	20	11.2	0	0			
**Follow-up**					6.813		0.078
Did not take place	57	32.0	37	20.8			
Took place	121	68.0	141	79.2			

Table 3 Distribution of different tumor entities across both patient groups (n = 356)

**Tumor entities**		**n**	**%**
**Brain tumors**		68	19.1
	C71 Malignant neoplasms of the brain	60	16.8
	D32 Benign neoplasm, meninges unspecified	4	1.1
	D33 Benign neoplasm of brain and other parts CNS	4	1.1
**Brain metastases**		40	11.2
	C79.3 Secondary malignant neoplasm of brain and cerebral meninges	40	11.2
**Head and neck tumors**		44	12.4
	C01 Malignant neoplasm of base of tongue	2	0.6
	C02 Malignant neoplasm of other and unspecified parts of tongue	3	0.9
	C03 Malignant neoplasm of gum	4	1.2
	C04 Malignant neoplasm of floor of mouth	3	0.9
	C05 Malignant neoplasm of palate	1	0.3
	C06 Malignant neoplasm of other and unspecified parts of mouth	2	0.6
	C09 Malignant neoplasm of tonsil	6	1.7
	C10 Malignant neoplasm of oropharynx	6	1.7
	C11 Malignant neoplasm of nasopharynx	3	0.9
	C13 Malignant neoplasm of hypopharynx	2	0.6
	C15 Malignant neoplasm of esophagus	4	1.2
	C32 Malignant neoplasm of larynx	6	1.7
	C76.0 Malignant neoplasm of other and ill-defined sites: head, face, neck	1	0.3
	C80 Malignant neoplasm, without specification of site	1	0.3
**Bronchial carcinomas**		18	5.1
	C34 Malignant neoplasm of bronchus and lung	16	4.5
	C78 Secondary malignant neoplasm of respiratory and digestive organs	2	0.6
**Breast carcinomas**		62	17.4
	C50 Malignant neoplasm of breast	62	17.4
**Colorectal and anal cancer**		6	1.7
	C18 Malignant neoplasm of colon	1	0.3
	C20 Malignant neoplasm of rectum	4	1.2
	C21 Malignant neoplasm of anus and anal canal	1	0.3
**Gynecological cancer**		4	1.1
	C52 Malignant neoplasm of vagina	2	0.6
	C53 Malignant neoplasm of cervix uteri	2	0.6
**Prostate cancer**		12	3.4
	C61 Malignant neoplasm of prostate	12	3.4
**Urinary tract cancer**		4	1.1
	C64 Malignant neoplasm of kidney, except renal pelvis	2	0.6
	C67 Malignant neoplasm of bladder	2	0.6
**Bone metastases**		42	11.8
	C79.5 Secondary malignant neoplasm of bone and bone marrow	42	11.8
**Lymphomas**		18	5.1
	C81 Hodgkin lymphoma	6	1.7

	C83 Non-follicular lymphoma	6	1.7
	C84 Mature T/NK-cell lymphomas	2	0.6
	C85 Other and unspecified types of non-Hodgkin lymphoma	4	1.2
**Leukemia**		6	1.7
	C91 Lymphoid leukemia	5	1.4
	C92 Myeloid leukemia	1	0.3
**Multiple myeloma**		6	1.7
	C90 Multiple myeloma and malignant plasma cell neoplasms	6	1.7
**Melanomas**		8	2.2
	C43 Malignant melanoma of skin	4	1.1
	C44 Other malignant neoplasms of skin	4	1.1
**Secondary, unspecified neoplasms of lymph node**		10	2.8
	C77 Secondary and unspecified malignant neoplasm of lymph nodes	10	2.8
**Carcinoma of unknown primary**		2	0.6
	C80 Malignant neoplasm, without specification of site	2	0.6
**Others**		6	1.7
	C37 Malignant neoplasm of thymus	2	0.6
	D35 Benign neoplasm of other and unspecified endocrine glands	4	1.2
**Total**		356	100

Table 4 Distribution of foreign patients' nationalities

	n	% of subpopulation	% of total
**Eastern Europe**			
Russian	13	24.5	7.3
Hungarian	6	11.3	3.4
Romanian	6	11.3	3.4
Polish	5	9.4	2.8
Bulgarian	4	7.5	2.2
Bosnian	3	5.7	1.7
Georgien	3	5.7	1.7
Ukrainian	3	5.7	1.7
Serbian	2	3.8	1.1
Belarusian	1	1.9	0.6
Czech	1	1.9	0.6
Kazakh	1	1.9	0.6
Kosovo	1	1.9	0.6
Macedonian	1	1.9	0.6
Moldavian	1	1.9	0.6
Slovenien	1	1.9	0.6
Yugoslavian	1	1.9	0.6
**Total**	**53**	**100.0**	**29.8**
**Southern Europe/Turkey**			
Turkish	28	52.8	15.7
Italian	12	22.6	6.7
Armenian	3	5.7	1.7
Portuguese	2	3.8	1.1
Greek	4	4.5	2.2
Spanish	4	4.5	2.2
**Total**	**53**	**100.0**	**29.8**
**Middle East**			
Saudi-Arabian	10	29.4	5.6
Syrian	8	23.5	4.5
United Arab Emirates	8	23.5	4.5
Iranian	3	8.8	1.7
Iraqi	3	8.8	1.7
Afghan	1	2.9	0.6
Kuwait	1	2.9	0.6
**Total**	**34**	**100.0**	**19.1**
**North, Western, and Central Europe**			
French	5	26.3	2.8
Belgian	3	15.8	1.7
Dutch	3	15.8	1.7
British	2	10.5	1.1
Danish	1	5.3	0.6
Finnish	1	5.3	0.6
Lettland	1	5.3	0.6
Luxembourg	1	5.3	0.6
Swedish	1	5.3	0.6
Swiss	1	5.3	0.6
**Total**	**19**	**100.0**	**10.7**
**Africa**			
Lybian	3	27.3	1.7
Moroccan	2	18.2	1.1
Congolese	1	9.1	0.6
Namibian	1	9.1	0.6
Somalian	2	18.2	1.1

	Togo	1	9.1	0.6
	Tunesian	1	9.1	0.6
**Total**		**11**	**100.0**	**6.2**
**North and South America**				
	American	2	66.7	1.1
	Canadian	1	33.3	0.6
**Total**		**3**	**100.0**	**1.7**
**Asia**				
	Filipino	2	40.0	1.1
	Thai	2	40.0	1.1
	Pakistani	1	20.0	0.6
**Total**		**5**	**100.0**	**2.8**
**Total**		**178**		**100.0**
